# The Relations between Television Exposure and Executive Function in Chinese Preschoolers: The Moderated Role of Parental Mediation Behaviors

**DOI:** 10.3389/fpsyg.2017.01833

**Published:** 2017-10-17

**Authors:** Xiaohui Yang, Zhe Chen, Zhenhong Wang, Liqi Zhu

**Affiliations:** ^1^CAS Key Laboratory of Behavioral Science, Institute of Psychology, Chinese Academy of Sciences, Beijing, China; ^2^School of Psychology, Shaanxi Normal University, Xi’an, China; ^3^Department of Human Ecology, University of California, Davis, Davis, CA, United States

**Keywords:** television exposure, executive function, parental mediation, preschoolers

## Abstract

The present study examined the relations between preschoolers’ television exposure and executive functions (EF). One hundred and nineteen 3- to 6-year-old children and their parents participated. Parents filled in a questionnaire regarding children’s television viewing time, television content and parental mediation behaviors about their child’s television viewing. The children were asked to finish six EF tasks, including the backward digit span task, the spatial span task, the boy–girl Stroop, the Simon task, the flanker task and the Tower of Hanoi task that assessed working memory, inhibition and planning, respectively. Children’s vocabulary was tested using Peabody Picture Vocabulary Test, and included as control variables in addition to socioeconomic status of the participated families. The results showed that television viewing time and child-directed educational programs were positively associated with EF. In addition, television content fully mediated the effect of television viewing time on EF and parental restrictive approach strategies moderated the effect of television viewing time on EF.

## Introduction

With the development and widespread use of electronic media, children now are using electronic media more and more, often at a very early age. Electronic media may be especially influential to children during periods of rapid brain development and plasticity (Christakis et al., 2012; Bush and Boyce, 2014) and may have powerful impacts on the development of social emotional competencies (e.g., Babaroglu, 2013) and cognitive capacities including executive function (EF) (e.g., Lillard and Peterson, 2011; Nathanson et al., 2014). EF refers to a family of top-down mental processes that aid in the monitoring and control of thought and action, and develops at a fast rate during the preschool period (Miller and Cohen, 2001). EF skills are essential for success in school and in life; and cognitive, social, and psychological development (Diamond, 2013). Many studies have addressed the possible effects of television exposure on children’s EF (e.g., Barr et al., 2010; Wartella et al., 2010; Lillard and Peterson, 2011; Nathanson et al., 2014) because television is the most prominent media in the lives of young children. However, the results of these many studies have been contradictory: Some have reported negative effects of television viewing on children’s EF (Barr et al., 2010; Lillard and Peterson, 2011; Nathanson et al., 2014), while others have found that children’s television viewing facilitated their EF (Linebarger et al., 2014). It has recently been suggested that the potential effects of media use depend not only on what is watched but also how it is watched (McHale et al., 2009; Connell et al., 2015). In the context of children’s media use, parental mediation behaviors regarding children’s media use may play a critical role in moderating the effect of media use on children’s EF.

While studies of EF development have focused on American or European children, few studies have examined the relation between television exposure and EF in other cultures, including Chinese culture. On the one hand, television programs in China and the amount of time that Chinese children spend watching television may be different from other cultures. In addition, unlike other western nations that provide guidelines about young children’s screen media use, there are no official recommendations regarding children’s digital media use or television viewing in China. On the other hand, Chinese children perform better than their western counterparts on EF tasks (Sabbagh et al., 2006). This may be partly because Chinese parents (Chao and Tseng, 2002) and teachers (Wang and Mao, 1996; Lan et al., 2009) request for self-control in everyday conduct. Investigating the relations between television exposure and the development of EF among Chinese preschoolers thus allows the examination of cultural similarities and differences in comparison with the results already reported in the literature.

Various theories have been proposed to explain why media exposure including television viewing may influence child development (Nikkelen et al., 2014). There are several potential ways that television exposure may impact child development outcomes. The first involves television watching time, which may displace activities such as reading or sleeping (Anderson et al., 2001). Still, previous research suggests that media use typically displaces functionally similar activities rather than activities that are more educationally valuable (Kirkorian and Anderson, 2009). The second way involves the content of television programs. Violent media may lead to ADHD-related behaviors (such as attention problems, hyperactivity, and impulsivity) by its effect on children’s arousal system (Bushman and Huesmann, 2006) or by activate a violence script (Anderson and Bushman, 2001). Other researchers suggest that the fast-paced media, by the frequent use of cuts and edits, built the child an attentional style of scanning and shifting (Jensen et al., 1997) which may interfere with the development of attentional capacities in tasks that require sustained attention (Christakis, 2009). The third way is about children’s media use context such as parental guidance behaviors which can moderate the effect of media use.

**FIGURE 1:**
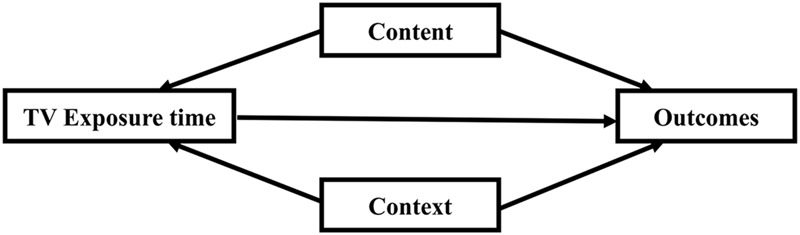
Conceptual model of television exposure and child outcomes, Christakis (2009).

**FIGURE 2:**
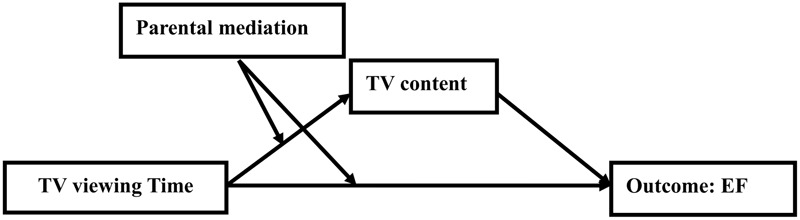
The proposed conceptual model of effects of television exposure on EF.

Christakis (2009) proposed a conceptual model (**Figure 1**) to understand the effect of television exposure on child development outcomes. This model, which can also help analyze and explain the effect of television exposure on EF, suggests that *what* children watch (content) and *how* they watch (context) as well as how much they watch are important factors influencing the nature of television exposure effects (Nikkelen et al., 2014). Most previous studies focused on the direct effect of television exposure time or viewing content on child development (e.g., Wright et al., 2001; Nathanson et al., 2014). Recent study revealed television viewing context as important factors which can moderate the effect of television exposure (Linebarger et al., 2014). Based on the model posited by Christakis (2009), the current study was designed to examine the effect of three aspects of television exposure on Chinese preschoolers’ EF: *television viewing time, television content*, and *parental mediation behaviors* on child’s television viewing. We developed a moderated mediation model (**Figure 2**) by which we examined both the unique effects, namely, time and content, and the indirect effect (mediation effect) between these factors and children’s EF, moderated by parent mediation behaviors impacting their children’s television viewing to obtain a comprehensive understanding of the influence of television exposure. “A mediating variable transmits the effect of an independent variable on a dependent variable” (Mackinnon et al., 2007). Thus, the mediation analysis will help to identify potential processes between the independent variable and the dependent variable. Moderator is a variable that affects the strength of the relation between two variables. Moderator is usually an interaction, the relation between independent variable and dependent variable depends on a third variable (Mackinnon et al., 2007). Thus, the moderation analysis will help to answer how the relations between independent variable and dependent variable would change under different contexts. In addition, if a mediation effect depends on a third variable, the mediation effect was moderated and the moderated mediation analysis is needed. In other word, the mediation effect cannot remain constant across different contexts, groups of individuals, or values of the independent variable (Preacher et al., 2007).

Much research on the effect of television exposure has focused on attention, which is the vital foundation of EF processes (Posner and Rothbart, 2007). Yet these studies have reported mixed results (Christakis et al., 2004; Stevens and Mulsow, 2006; Foster and Watkins, 2010). Most of the published studies suggested that television viewing time is associated with lower attention skills (Landhuis et al., 2007; Swing et al., 2010). However, other studies revealed little effect of television viewing on attention problems (Stevens and Mulsow, 2006; Foster and Watkins, 2010). Similarly, research on relations between overall time spent viewing television and EF also showed mixed results: Nathanson et al. (2014) found that television viewing time was negatively related to EF, while Linebarger et al. (2014) found that low-risk preschoolers demonstrated higher EF with increased television viewing time. This inconsistency may be due to several issues. First, the relationship between television viewing time and EF was curvilinear, demonstrating that moderation level of viewing was positively associated with academic achievement, while additional viewing had an increasingly negative impact on achievement (Williams, 1982; Razel, 2001). Secondly, much of existing studies, a single indicator was used to measure television exposure without distinguishing between different types and dimensions of television program content.

Previous research has also indicated that both the age when children start watching television and television content matter. Numerous studies revealed educational programs such as Sesame Street appear to be beneficial for young viewers’ language development and school readiness (e.g., Wright et al., 2001). Barr et al. (2010) found that children who watched higher amounts of adult-directed programs at age 1 showed poorer EF than those who started at age 4; in contrast, exposure to child-directed television programs at either age 1 or 4 was not associated with EF at age 4. Lillard and Peterson (2011) reported that fast-paced cartoons viewing would weaken the EF performance of 4 years old children. Their further research (Lillard et al., 2015a,b) revealed that fantastical content, not fast pacing, disrupted preschooler’ EF. However, Linebarger et al. (2014) showed that low-risk preschoolers demonstrated better EF with increasing exposure to non-educational television (both child-directed entertainment and adult-directed content). On the other hand, Nathanson et al. (2014) found that PBS viewing was positively related to EF while educational cartoon viewing was related to poorer EF performance. The possible reason for this inconsistency in the relation between television content and EF may be the somewhat different criteria used to categorize television content. In Linebarger et al. (2014) study, they used an established protocol to code the reported programs. While, Nathanson et al. (2014) used principal component analysis to get the children’s genre viewing. Additionally, the influence of television content and viewing time on young children’s EF may moderated by other factors, such as the context of children’s television viewing.

Parents play a crucial role in moderating the effects of television. Previous studies on parental mediation behaviors about children’s media use (Valkenburg et al., 1999) have defined three distinct strategies: restrictive mediation, coviewing, and instructive mediation. Restrictive mediation includes parents’ rules about the amount viewing time, permissible or forbidden types of content, and the use of viewing as a behavioral reward or punishment. Parent–child coviewing is defined as a shared set of motivations for viewing. Instructive mediation refers to an effort to discuss television content with children (Valkenburg et al., 1999). Studies have revealed that television co-use was associated with positive outcomes, including improves in comprehension and increased enjoyment of television content among preschoolers (Salomon, 1977), as well as increased attention and responsiveness among toddlers (Barr et al., 2008). In addition, parental mediation behaviors are associated with less children’s television viewing time (Bulck and Bergh, 2000; An and Lee, 2010; Wu et al., 2014). Linebarger et al. (2014) found that parenting style moderated the relation between children’s television exposure and EF. However, studies have not examined whether and how the associations between television exposure and children’s EF may be moderated by parental mediation which was the more specific aspect of parenting style about children’s media use. To address this issue, we tested whether the relation between television viewing and EF would be moderated by parental mediation.

In sum, the relationship between television viewing and EF is not well understood. To address this point, we conducted a comprehensive survey and laboratory measurement in 3- to 6-year-old children. Specifically, we aimed to examine how EF is associated with children’s television viewing time (overall amount of television viewing) and viewing of different content (i.e., adult-oriented and child-oriented television), and to explore the moderating role of parental mediation in these relationships. When measuring television content, we differentiated between adult-directed content and child-directed content, and regarding child-directed content, we further differentiated different kinds of programs, such as educational content, recreational content, and violent content. Last and most important, we extended Christakis’ (2009) model by proposing that television content would mediate the effect between television viewing time and EF. Given that viewing time always involves some type of content, the effect of viewing time would then be at least partly mediated by this content. The conceptual model of the present study is illustrated in **Figure 2**.

We formulated and tested the following hypotheses:

(1) Time spent television viewing will positively correlate with EF (H1);
(2) Television content will correlate with EF. Specifically, child-directed educational programs will positively correlate with EF (H2a) child-directed fast paced or violent programs (H2b) as well as adult-directed programs will negatively correlate with EF (H2c);
(3) Television content will mediate the effect of television viewing time on EF (H3);
(4) Parental mediation will moderate the direct effect of television viewing time on EF (H4a), and will moderate the indirect effect of television viewing time on EF through television content (H4b).

## Materials and Methods

### Participants

Participants were recruited from one preschool in a large city in northern China. A total of 119 preschoolers (51% girls) age range from 3.17 to 6.31 years old (*M* = 4.64 years old, *SD* = 0.92) and their parents (73% mothers) participated in this study. Participants are Han Chinese and speak Mandarin. We adopted two widely used markers of SES: maternal education and household income. 21.8% mothers held a graduate degree, 67.2% mothers were college educated, and 11% mothers indicated they had completed high school. Parents demonstrated their average monthly household income by choosing one of seven income levels, ranging from “less than RMB3,000” to “RMB30,000 or more.” The largest percentage of participants selected “RMB3,000 to RMB6,000” (33.6%), followed by “RMB6,000 to RMB8,000” (31.9%), “RMB8,000 to RMB10,000” (16.8%), “RMB10,000 to RMB15,000” (11.8%), “RMB15,000 to RMB30,000” (2.5%), and “less than RMB 3,000” (3.4%). The average annual household income of Chinese was 49,318 RMB in 2014 (He, 2015). Most families’ household incomes in the present study were above the national average income. Most of the families (79%) owned one television, and some of the families (18%) had more than two televisions, however, none placed televisions in children’s bedrooms.

### Procedure

This study was carried out in accordance with the ethical standards of Declaration of Helsinki and was approved by the Research Ethics Board of the Institute of Psychology, Chinese Academy of Sciences. Children were recruited using simple random sampling method based on the name list provided by the preschool. Parents were given consent forms and finished a questionnaire including their demographics and the television use of the participating child as well as parental guidance patterns on children’s television use. Children were interviewed one by one in a quiet room at their preschool by three trained researchers. The experimenters administered EF tasks and a vocabulary assessment during three sessions, each lasting between 10 and 20 min to avoid that children cannot concentrate on the tasks. All three sessions were done in the same day. The tasks in each session were in a fixed order. The order in which children completed the three test sessions was randomly assigned to eliminate possible order effects. Session 1 included the backward digit span task, the spatial span task and Peabody picture vocabulary test. Session 2 included the boy–girl Stroop, Simon task, and flanker task. Session 3 included the tower of Hanoi task. We gave each participated child a small gift and each parent some gifts for their participation.

### Children’s Television Exposure Measures

#### Television Viewing by Children

Parents estimated the hours their child watched television on an average weekday and on an average weekend day during three time periods, namely, morning, midday, and evening, respectively. Parents were told that viewing estimates should include “programs seen on a television, computer, or via DVD or a portable electronic device.” (Nathanson et al., 2014) The sum for weekday viewing was then multiplied by 5 and the sum for weekend viewing was multiplied by 2. We then summed the two products and divided it with 7 to generate average daily viewing in hours (*M* = 1.22 h, *SD* = 0.93).

#### Age of Starting to Watch Television

Parents reported the age at which their child first began watching television (Nathanson et al., 2014), choosing their answer on a scale from “never done,” “before 6 months old,” “between 6 months old and 1 year old,” “1 year old,” “2 years old,” “3 years old,” “4 years old” to “at or after 5 years old.”

#### Children’s Channel Viewing

In China, China Central Television (CCTV) and local television stations both have special channels to broadcast children’s programs. The highest ratings and the most popular children’s channels include CCTV-14 Children’s Channel, Golden Eagle Cartoon Channel of Hunan TV, and KAKU Children’s Channel of Beijing TV. On a scale from 0 (*never*) to 3 (*all of the time*), parents reported how often their child watched six different channels when they watched television, including CCTV-14 Children’s Channel, Golden Eagle Cartoon Channel, KAKU Children’s Channel, other cartoon channels, CCTV (other than the children’s channel), and other channels. A principal-components factor analysis with oblimin rotation was used to explore the factors of children’s channel viewing. Two factors were extracted, explaining approximately 58.24% of variance. The first factor consisted of the viewing of CCTV-14 Children’s Channel, KAKU Children’s Channel, Golden Eagle Cartoon Channel, and other cartoons channels, explaining about 35.5% of the variance. The second factor included viewing of CCTV (other than the children’s channel) and other channels, and explained 22.75% of the variance. Two factors were constructed into two scales to represent *child-directed programs viewing* and *adult-directed programs viewing*. The Cronbach’s alpha coefficients of the two scales were 0.70 and 0.65.

#### Children’s Genre Viewing

Based on the categories used in a previous study (Nathanson et al., 2014), parents reported how often their child watched various types of entertainment or educational content when they watched television. Using a scale from 0 (*never*) to 3 (*all of the time*), these program categories included “action cartoons (e.g., Kung Fu Panda, Ultraman),” “classic cartoons (e.g., Tom and Jerry),” “live educational children’s programs (e.g., Tree of Knowledge),” “fast-paced cartoons (e.g., Phineas and Ferb, Sponge Bob Square Pants),” “situation comedies for children (e.g., Star Elves Lan Duoduo, Balla Balla Little Magic Fairies),” and “educational cartoons (e.g., Dora the Explorer, Rainbow Cat, Blue Rabbit).”

#### Parental Mediation Behaviors on Children’s Television Use (Bulck and Bergh, 2000)

Parents were asked to indicate their mediation behavior regarding to children’s television viewing. They were asked to choose one of four answers (*never, seldom, sometimes*, and *often*) to each of 12 questions representing three types of parental mediation approaches. The three types of parental approaches included: (a) Instructive approach (e.g., “Tell my child when somebody in a show does something that is bad”); (b) Coviewing approach (e.g., “Watching television together with my child because it is important to her or him to do this activity together with me”); and (c) Restrictive approach (e.g., “Not allow my child to watch a certain program”). In this study, the Cronbach’s alpha coefficient of the three sub-scales were 0.80, 0.69, and 0.68.

### Child Measures of EF

Executive functions tasks were selected based on evidence for their reliability and appropriateness for use with preschoolers. EF was viewed as a set of complex cognitive skills involved in controlling, directing, and planning cognitive activities and behaviors (Diamond, 2013). In the present study, children’s working memory, inhibition, and planning were measured to represent the different aspects of EF development. In addition, these EF components are highly correlated and are essentially a single component during preschool years (Wiebe et al., 2008) and aggregated measures of EF are stable and robust (Willoughby et al., 2011). Based on previous study (Nathanson et al., 2014), scores on all the measured EF tasks were standardized to *Z*-scores and then summed to create an overall assessment of children’s EF. Higher scores of EF reflect better abilities of EF.

#### Working Memory

The backward digit span task (Carlson et al., 2002) and the spatial span task were used to test children’s working memory. In the backward digit span task, the child was told a series of digits and asked to repeat the digits in reverse. Children received three trials each of two-, three-, and four-digit lengths. The tasks were ended when children gave wrong answers on two trials of a given length. Performance was measured with the highest digit- length completed. In the spatial span task, a little chicken appeared on the computer screen at different locations in a 3 × 3 grid, with each appearance remaining on the screen for 1000 ms. Children were instructed to reproduce the sequence of the locations by touching the grids. There were three trails in each level. The task was ended if children failed more than twice on one given level. Performance was measured with the highest level completed.

#### Inhibition

Three tasks were used to assess different aspects of inhibition. In Boy–Girl Stroop task (Miller et al., 2012) children were instructed to say “boy” when they see a cartoon girl on the screen and to say “girl” when seeing a cartoon boy on the screen. The task included 20 pictures, with each type of picture appearing 10 times. Performance was measured with the number of times children correctly labeled each picture. In the Simon task (Davidson et al., 2006), a color picture of either a frog or butterfly was presented on the left or right side of the computer screen. The appearance of frog was always associated with right key press response and the butterfly was associated with left key press response. The stimuli were presented randomly on the left or right of the screen over the block of 20 trials, yielding congruent and incongruent trials. In the Flanker task (Fan et al., 2002), children were presented with a row of five fish with the target fish flanked on each side by two fish facing the same or the opposite direction (congruent or incongruent conditions, respectively). Children need to identify, the direction the target fish was facing on each trail by key press. There were two blocks each consisted of 20 trials and the target stimuli were presented randomly facing left or right.

#### Planning

The Tower of Hanoi task (ToH) (Lillard and Peterson, 2011) involved a base with three long pegs and a larger and a smaller disk that fit on the pegs, and a picture portrayal a goal state. A story about helping monkeys home was introduced to help the child to understand the rules of this task: only 1 disk could be moved at a time, the disks always needed to stay on pegs, and the bigger disk could never go on top of the smaller disk. The children were given a score of 1 if they moved the disks successfully by following all the rules. Children who broke a rule or failed to complete the task were given a score of 0.

### Covariates

Children’s age, gender and family SES have been linked to differences in the amount and quality of television exposure (Anderson et al., 2001; Zimmerman and Christakis, 2007) as well as the development of EF (Linebarger et al., 2014) were as covariates in all models. Children’s vocabularies were also controlled as covariate because children with higher language ability may perform better on EF tasks due to their better understanding the task instructions (Bernier et al., 2010). Children’s receptive vocabulary was assessed with the Peabody picture vocabulary test – Chinese edition (PPVT-C) (Sang and Miao, 1990). The experimenter stated a word, and children had to point to the corresponding picture out of four choices. The task ended when children made more than five errors on a set of eight words. Performance was measured in terms of raw scores (i.e., ceiling item – total errors).

### Analysis Plan

Multiple hierarchical linear regression models were computed with SPSS 19.0 to test the direct effect of television viewing time, viewing content and parental mediation on EF (Hypotheses 1 and 2). SPSS macro (PROCESS; Preacher et al., 2007; Hayes, 2015) was run to test the moderated mediation model (Hypotheses 4). PROCESS is a bootstrapping procedure, which provide the estimates of the confidence intervals (CIs) of the indirect effect or conditional indirect effect from bootstrapped samples. Conditional indirect effect is the magnitude of an indirect effect at a particular value of the moderator (Preacher et al., 2007). If the conditional indirect effects at levels moderator were significantly different, then the mediation effect was moderated. In order to test the significance of the moderated mediation effect, Hayes (2015) proposed a term called index of moderated mediation. The index of moderated mediation is a quantification effect of the moderator on the indirect effect of independent variable on dependent variable through the mediator (Hayes, 2015). If the 95% CI of the index did not contain zero, the moderated mediation was significant. A bootstrapping approach provides much greater statistical power for mediation analysis than the traditional causal steps approach (Hayes, 2009). Variance inflation factors (VIF) was used to diagnose multicollinearity of these models. Due to the non-normal distributions of children’s television viewing time (skewness = 1.88, kurtosis = 6.29), a square-root transformation was conducted to make the distributions more normal. This strategy was used by previous researches to deal with the media use variables (e.g., Ferguson and Brent, 2014).

## Results

The descriptive statistics for all the measures are displayed in **Table 1**.

**Table 1:** Descriptive analysis of the variables.

Variables	*M*	*SD*	Min	Max
Child’s age (years)	4.64	0.92	3.17	6.31
Maternal education	3.11	0.56	2	4
Family income	3.08	1.16	1	6
Child vocabulary	59.17	31.27	9	134
TV viewing (hours)	1.22	0.93	0	6.14
Onset of TV viewing (years)	1.36	0.80	0.5	4.0
Child channels viewing	1.39	0.71	0	3
Adult channels viewing	0.51	0.46	0	2
Action cartoons viewing	0.81	0.78	0	3
Classic cartoons viewing	1.35	0.70	0	3
Children’s live show programs viewing	1.19	0.83	0	3
Fast-paced cartoons viewing	1.25	0.82	0	3
Educational cartoons viewing	1.29	0.93	0	3
Children’s situation comedies viewing	0.53	0.72	0	3
Restrictive approach	3.23	0.65	1	4
Coviewing approach	3.23	0.58	1	4
Instructive approach	3.31	0.65	1	4
Back-digit span	1.84	0.83	1	5
Spatial span task	3.12	1.12	1	6
Boy–girl stroop	10.47	5.83	0	20
Tower of Hanoi	0.36	0.53	0	2
FL-in- acc	0.72	0.26	0.25	1.00
SI-in-acc	0.83	0.17	0.25	1.00
EF	0	4.30	-9.83	9.31

*Categories for parent education were 1 = less than junior high school, 2 = high school, 3 = undergraduate, 4 = graduate degree; Categories for family income 1 = less than 3,000 Yuan, 2 = 3,000 ~ 6,000 Yuan, 3 = 6,000 ~ 8,000 Yuan, 4 = 8,000 ~ 10,000 Yuan, 5 = 10,000 ~ 15,000 Yuan, 6 = 15,000 ~ 30,000 Yuan, 7 = more than 30,000 Yuan. FL-in-acc = the accuracy of incongruent trials in Flanker task; SI-in-acc = the accuracy of incongruent trials in Simon task; EF was the composite *Z*-scores of six tasks.*

**Table 2** presents the bivariate correlations between non-media variables, media exposure variables and EF. Children’s EF was highly positively correlated with their age and vocabulary. Positive correlations were evident among television viewing time, children’s channels and cartoon programs. Moreover, the television viewing time was not significantly correlated with EF. While, child’s channels viewing and certain types of cartoon programs including classic cartoons viewing, fast-paced cartoons viewing, and children’s situation comedies viewing were positively correlated with EF. These zero-order correlations should be interpreted with caution. For instance, older children may watch more television and have better EF development than younger children. The results indeed revealed that child’s age was positively related with child’s channels viewing and certain types of cartoon programs. Thus, further regression analysis with these confounding variables under control was conducted.

**Table 2:** Bivariate correlations among media variables, non-media variables, and EF.

	1	2	3	4	5	6	7	8	9	10	11	12	13	14	15	16	17	18
(1) EF	-																	
(2) Child’s age	0.79**	-																
(3) Child’s gender	0.04	0.10	-															
(4) Child’s vocabulary	0.72**	0.76**	0.12	-														
(5) Maternal education	0.03	0.11	-0.04	0.22*	-													
(6) Family income	0.14	0.20*	0.07	0.12	0.30**	-												
(7) Onset of TV viewing	0.17	0.20*	-0.05	0.12	0.02	-0.04	-											
(8) TV viewing time	0.12	0.04	0.02	-0.08	-0.02	-0.01	-0.20*	-										
(9) Child channels viewing	0.21*	0.22*	-0.06	0.11	0.02	0.01	-0.05	0.43**	-									
(10) Adult channels viewing	0.11	0.05	-0.12	0.08	-0.05	-0.07	0.03	0.22*	0.17	-								
(11) Action cartoons viewing	0.16	0.22*	0.24**	0.13	0.09	0.08	-0.03	0.12	0.14	-0.09	-							
(12) Classic cartoons viewing	0.30**	0.13	-0.09	0.10	0.12	0.07	-0.12	0.33**	0.35**	0.05	0.30**	-						
(13) Live educational children’s programs viewing	0.14	-0.01	-0.22*	-0.02	0.08	-0.07	0.03	0.17	0.28**	0.30**	0.06	0.35**	-					
(14) Fast-paced cartoons viewing	0.29**	0.20*	0.03	0.22*	-0.02	0.05	-0.07	0.39**	0.55**	0.18	0.23*	0.38**	0.25**	-				
(15) Educational cartoons viewing	0.04	-0.04	-0.23*	-0.01	0.05	-0.02	-0.04	0.29**	0.42**	0.02	-0.05	0.40**	0.53**	0.42**	-			
(16) Situation comedies for children viewing	0.24*	0.24**	-0.15	0.10	0.13	0.07	0.05	0.25**	0.36**	0.29**	0.14	0.10	0.43**	0.33**	0.40**	-		
(17) Restrictive approach	-0.16	-0.04	-0.01	0.01	0.16	0.09	0.13	-0.15	-0.05	0.01	-0.11	-0.13	-0.01	-0.14	0.07	0.02	-	
(18) Coviewing approach	-0.07	-0.13	-0.10	-0.17	-0.16	-0.19*	0.13	0.02	-0.07	0.00	-0.05	-0.06	0.11	-0.03	0.05	0.12	0.33**	-
(19) Instructive approach	0.13	0.04	-0.02	0.02	-0.16	-0.03	0.09	-0.01	0.08	0.02	0.12	0.10	0.23*	0.03	0.12	0.21*	0.16	0.38^∗∗^

*^∗^*p* < 0.05, ^∗∗^*p* < 0.01.*

Four separate hierarchical regression models for each set of media exposure variables were conducted to test hypotheses 1 and 2: the direct effect of television viewing time, viewing content and parental mediation (**Table 3**). In each model child’s age, gender, vocabulary, maternal education and family income were as control variables entered in first step. Children’s television viewing time and the age start watching television, children’s channel viewing, children’s genre viewing and parental guidance patterns on children’s television use, were entered in second steps of *Model 1~Modle 4* separately. These models allowed us to observe the unique contribution of each set of media variables to the variance of EF above and beyond the control variables. VIFs of the four models ranged from 1.03 to 2.60, which were lower than the recommended level (VIF < 10) (Hair et al., 1995) suggesting that these models did not violate the stability of the parameter estimates. Across the four models, child’s age, gender, vocabulary, maternal education, and family income accounted for a significant 67% of the variance in EF with child’s age (β = 0.55, *p* < 0.001), vocabulary (β = 0.33, *p* < 0.001) and mother education (β = -0.12, *p* < 0.05) as significant predictors. In *Model* 1, Children’s television viewing time and the age start watching television explained a significant 2% of additional variance with television viewing time related to better EF, Cohen *f*^2^ = 0.06. In *Model* 2, children’s channel viewing did not account for significant variance in EF, Cohen *f*^2^ = 0.01. In *Model* 3, six kinds of children’s cartoons or programs explained a significant 7% of additional variance in EF, Cohen *f*^2^ = 0.27. Classic cartoons viewing was significantly positively related with EF. Live educational children’s programs viewing was marginal significantly positively related with EF (β = 0.12, *p* = 0.065). While, educational cartoons viewing was marginal significantly negatively related with EF (β = -0.13, *p* = 0.056). In *Model* 4, three kinds of parental guidance patterns account for a significant 3% of additional variance in EF with restrictive approach significantly related with poor EF, Cohen *f*^2^ = 0.1. Cohen’s categories about effect size (Cohen, 1977) indicates that effect size of *f*^2^ smaller than 0.02 is small effect, 0.15 is moderate effect and 0.35 means large effect. The effect size of television viewing time account for EF was small to medium effect and the effect size of viewing content was medium to large effect.

**Table 3:** Summary of hierarchical regression analyses for non-media and media variables predicting children’s EF.

	Variables	*B*	*SE*	β	Unique *R*^2^	*F* change	Cohen *f*^2^
Model 1	Onset of TV viewing	0.31	0.30	0.06	0.02	3.60*	0.06
	TV viewing time	0.58	0.22	0.15**			
Model 2	Child channels viewing	0.27	0.34	0.05	0.004	0.708	0.01
	Adult channels viewing	0.40	0.52	0.04			
Model 3	Action cartoons viewing	-0.49	0.32	-0.09	0.07	4.42***	0.27
	Classic cartoons viewing	1.44	0.39	0.23***			
	Live educational children’s programs viewing	0.62	0.33	0.12^a^			
	Fast-paced cartoons viewing	0.23	0.32	0.04			
	Educational cartoons viewing	-0.61	0.32	-0.13^b^			
	Situation comedies for children viewing	0.42	0.38	0.07			
Model 4	Restrictive approach	-1.16	0.38	-0.18**	0.03	3.85*	0.1
	Co-using approach	0.59	0.47	0.08			
	Instructive approach	0.55	0.38	0.08			

*In each model child’s age, gender, vocabulary, maternal education and family income were as control variables entered in first step, *R*^2^ = 0.67, *p* < 0.001. ^∗^*p* < 0.05, ^∗∗^*p* < 0.01, ^∗∗∗^*p* < 0.001, ^a^*p* = 0.065, ^b^*p* = 0.056.*

SPSS macro PROCESS (Model 4) (Hayes, 2015) was run to test the multiple mediator model for Hypotheses 3. The results showed that the only indirect effect estimate for television viewing time on EF through classic cartoons viewing (*ab* = 0.24, *SE* = 0.11, 95% bias corrected CI [0.08, 0.53]) and live educational children’s programs viewing (*ab* = 0.10, *SE* = 0.06, 95% bias corrected CI [0.01, 0.29]) were significant because both 95% bias corrected CI did not contain zero. In each analysis, maternal education and family income, children’s age, gender, and vocabulary were controlled. The mediated model explained 69% variance in EF (*p* < 0.001). Number of bootstrap samples was 5000.

**FIGURE 3:**
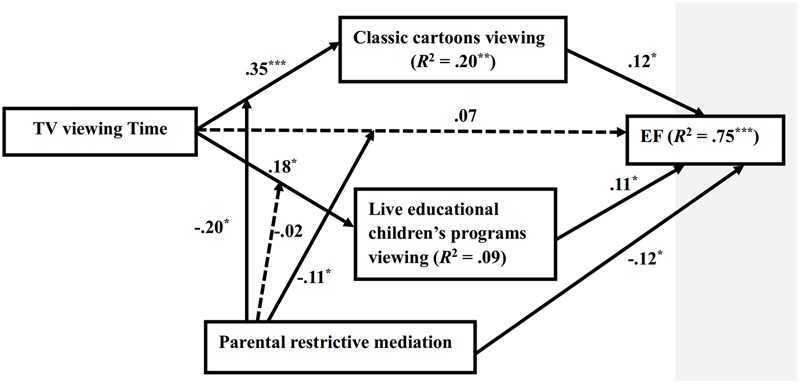
Summary of the moderated mediation model. Restrictive approach and television viewing time were mean centered. Child’s age, gender, vocabulary, maternal education, and family income were as control variables. ^∗^*p* < 0.05, ^∗∗^*p* < 0.01, ^∗∗∗^*p* < 0.001. Dotted lines indicate the coefficients were not significant.

We further applied the SPSS macro PROCESS (Model 8) (Hayes, 2015) to test if restrictive approach strategies can moderate the mediation model (Hypotheses 4). In each analysis, we controlled for maternal education and family income, children’s age, gender, and vocabulary. Number of bootstrap samples was 5000. Conditional direct effects and indirect effects of television viewing time on EF were tested at three levels of restrictive approach: the mean level of restrictive approach and one SD below and above the mean level. Result showed that firstly, restrictive approach strategies moderate the direct effect of television viewing time on EF. Television viewing time had a positive effect on EF only at the low level of restrictive approach (*B* = 0.76, *SE* = 0.33, 95% CI [0.10, 1.42]). Secondly, restrictive approach strategies moderated the indirect effect of television viewing time on EF through child classic cartoons viewing, with the index of moderated mediation equal to -0.17 (*SE* = 0.12, 95% CI [-0.47, -0.003]). The index was negative, meaning that the indirect effect of television viewing time on EF through child classic cartoons viewing was a decreasing function of restrictive approach strategies. Conditional indirect effect(s) of television viewing time on EF at values of the restrictive approach showed that only at a low to modest level of restrictive approach, there was a *positive* indirect effect of television viewing time on EF through child classic cartoons viewing (*ab* = 0.28, *SE* = 0.16, 95% bias corrected CI [0.05, 0.67] and *ab* = 0.17, *SE* = 0.08, 95% bias corrected CI [0.03, 0.42]). Thirdly, restrictive approach strategies did not moderate the indirect effect of television viewing time on EF through live educational children’s programs viewing, with the index of moderated mediation equal to -0.01 (*SE* = 0.07, 95% CI [-0.19, 0.11]), 95% CI of the indexes contain zero, suggesting that the tests of moderated mediation were not statistically significant. However, the indirect effect of television viewing time on EF through live educational children’s programs viewing was significant only at the modest level of restrictive approach (*ab* = 0.08, *SE* = 0.05, 95% bias corrected CI [0.01, 0.23]). Results about the moderated mediation model was presented in **Table 4** and **Figure 3**.

**Table 4:** Summary of the moderated mediation model.

	Mediator: Classic cartoons viewing			Mediator: Live educational children’s programs viewing			Outcomes: EF		
Variables	*B*	*SE*	β	*B*	*SE*	β	*B*	*SE*	β
Age	0.02	0.11	0.02	0.00	0.13	0.00	2.39	0.37	0.51***
Gender	-0.18	0.12	-0.13	-0.35	0.15	-0.22*	-0.35	0.44	-0.04
Child’s vocabulary	0.00	0.00	0.08	0.00	0.00	0.01	0.05	0.01	0.34***
Maternal education	0.13	0.12	0.11	0.15	0.15	0.10	-0.94	0.41	-0.12*
Family income	0.02	0.06	0.03	-0.06	0.07	-0.09	0.14	0.20	0.04
TV viewing time	0.22	0.06	0.35***	0.14	0.07	0.18*	0.27	0.21	0.07
Restrictive approach	-0.13	0.10	-0.12	0.00	0.12	0.00	-0.78	0.34	-0.12*
Classic cartoons viewing							0.75	0.35	0.12*
Children’s live educational programs viewing							0.55	0.28	0.11*
TV viewing time × Restrictive approach	-0.22	0.10	-0.20*	-0.02	0.12	-0.02	-0.75	0.35	-0.11*
*R*^2^	0.20**			0.09			0.75***		

*Restrictive approach and television viewing time were mean centered. ^∗^*p* < 0.05, ^∗∗^*p* < 0.01, ^∗∗∗^*p* < 0.001.*

## Discussion

The present study is one of few studies conducted to examine the relations between television viewing and cognitive outcomes among Chinese preschoolers. Most importantly, the study extends earlier work by considering multiple variables related to children’s television viewing behavior: how old they were when they started watching, how much time they watch (television viewing time), what they watch (television viewing content), and how they watch (television viewing context: parental behaviors related to children’s television viewing), as well as multiple measures of EF. Results of the study yielded significant implications for understanding what, how, and when television viewing affects the development of EF. In addition, the present results also suggest both cultural similarities and differences in the associated patterns reported in the literature.

The result that television viewing time was positively associated with EF confirms Hypothesis 1. Hypothesis 2 was also supported by the finding that although child-directed live educational television and classical cartoons viewing was positively associated with EF while adult-directed television and other kinds programs were not significantly correlated with EF. In addition, it is evident that child’s live educational program and classical cartoons viewing mediated the effect of television viewing time on EF, and these results confirm Hypothesis 3. Parental restrictive approach strategies were also found to moderate the direct effect of television viewing time on EF, as well as the indirect effect of television viewing time on EF through child classic cartoons. Hypothesis 4 which indicated that parental mediation can moderate the direct effect of television viewing on EF and can moderate the indirect effect of television viewing on EF through television content were confirmed. Our proposed conceptual model (**Figure 2**) was thus confirmed.

The positive association between television viewing time and EF evident in the present study is different from most previous studies, which showed negative or null associations between television viewing time and EF (Nathanson et al., 2014; Blankson et al., 2015). These inconsistencies in the nature of the relation between television viewing and EF may be explained with the following possibilities.

First, the relation between television viewing time and the development of EF appears to be a reverse U shape, instead of a linear function (Hofferth, 2010). A significant difference between the current and previous results is that the television viewing time of this Chinese sample was significantly shorter than their counterparts reported in other studies (*M* = 1.22 h, *SD* = 0.93 vs. *M* = 2.87 h, *SD* = 1.99) (Nathanson et al., 2014). Other previous investigation about Chinese preschooler’s media use showed similarity result that they spent about 90 min per day to watch television (Li et al., 2014). Foster and Watkins (2010) also suggested that the non-linear specification reveals an association between television watching and attention problems that exists only at very high levels of television viewing. Similarly, several other studies (Obel et al., 2004; Christakis, 2009) have shown a null relationship between television viewing and attention problems in low levels of viewing.

Second, what children watch (content) and how they watch (context) are arguably more important television viewing variables than how much children watch. As revealed in the present study, the effect of television viewing time on EF disappeared when television viewing content was included in the regression model, suggesting that television viewing content fully mediated the effect of television viewing time on EF. It is revealed that classical cartoons and live educational programs viewing were positively correlated with EF. There are many live educational programs such as tree of knowledge directed to preschoolers. These high-quality programs may enable children to develop EF skills.

Third, it is important to consider children’s media use context as the moderate effect of parental restrictive behaviors was evident in the present study. The design of our study enabled us to examine the relation between television viewing and EF while taking context variables into consideration, and our findings suggest a more complex picture: the relation patterns between television viewing and EF also depend on parental restrictive behaviors. Restrictive mediation includes parents’ rules regarding the amount of viewing, permissible and forbidden types of content, and the use of viewing as a behavioral reward or punishment. A significant positive relation between television viewing time and EF was evident only when restrictive mediation was at a low level. In addition, only when restrictive approaches were at low or moderate levels, there was a positive indirect effect of television viewing time on EF through child classical cartoon and live educational programs viewing. Combined with the results of bivariate correlation analyses, it is evident that a restrictive approach was negatively correlated with television viewing time and most kinds of child programs. Although the correlations were not significant, they suggest that parental restrictive behaviors tend to be associated with less television viewing.

A particularly interesting finding of this study was the main negative effect of parental restrictive approach strategies on EF. There are several possible explanations. A parental restrictive approach is associated with an arbitrary parenting style, which involves depriving children’s opportunities to regulate their own behavior and requiring them to adapt continually to another’s perspective (Talwar et al., 2011). Such an approach might be the cause of slow development of EF. An alternative explanation is that parents whose child demonstrates poor EF may set more limitations on their child’s television watching. The present study urges parents and educators to be aware of the potential moderation effect of media use guidance behaviors on children’s media use as well as their development. Parents should care about their arbitrary guidance behaviors about their children’s media use. Future work is needed to further explore the role of parental meditation behaviors related to children’s television viewing and its impact on EF.

Although not part of our major concern of the study, an unexpected result involving the relation between mother education and child’s EF. Contrary to prior study (Sarsour et al., 2011), we found mother education was negatively related to EF. This may partly due to the narrow range of mother education in this sample with most mothers hold university or college degree (67%) and postgraduate degree (22%).

In sum, the present study was conducted to examine the relations between children’s television viewing and the development of EF with various features and strengths. The quality of the measures is one advantage. Our EF tasks were measured directly by children’s responses instead of using parent report. Moreover, in the present study, we controlled other factors that could influence both children’s EF and television viewing. Finally, our research was guided by a theoretical framework and the results largely confirmed the proposed conceptual model. The findings of this study suggest that EF is associated with differences in both television viewing time and content. These findings form an important first step toward the conceptualization and investigation of more nuanced models on the relationship between television viewing and EF. Future research should not only distinguish between the different types of content that children are exposed to, but must also more systematically conceptualize and model mediating and moderating factors in the investigation of relations between EF and television. Building such indirect and conditional effects models will enable us to gain a deeper understanding of the relationship between media use and EF.

Although our findings contribute to our understanding of the relation between young children’s television viewing and the development of EF, there are still limitations in our study. First, as with most previous studies, the cross-sectional nature of this study prevents us from drawing conclusions about the direction and causality of the current results. Although television viewing might well affect the development of EF, we cannot exclude the possibility that children with higher EF ability are more capable of choosing child directed educational television programs and spend more time watching them (Wright et al., 2001). Research that further explores the bidirectional relationships between EF and television viewing is therefore urgently needed. Another major limitation of the present study is that the sample overall watched relatively little television. Further study with the nationally representative sample to examine the relations between television viewing and child cognition development is needed. Finally, the present study mainly focused on the effect of television exposure. Given the rapid rise in the use of mobile technology by young children, future studies should consider addressing issues related to relations between children’s mobile media use and higher-order cognitive abilities.

## Conclusion

This study reveals that television exposure is related to children’s EF. Furthermore, the relation between television viewing and EF development depend on programming content and parents’ media monitoring behaviors. Given the important role of EF in child’s social function development and school achievement, as well as the widely use of electronic media by young children, increasing our understanding of the links between children’s television viewing as well as the new interactive media and EF is urgently needed.

## Author Contributions

XY designed the study, collected and analyzed data, and drafted the paper. LZ co-designed and revised the paper critically. ZC and ZW revised the paper critically.

## Conflict of Interest Statement

The authors declare that the research was conducted in the absence of any commercial or financial relationships that could be construed as a potential conflict of interest.
